# Supplementary material for: ChIP‐Atlas: a data‐mining suite powered by full integration of public ChIP‐seq data
Source: EMBO Rep. 2018 Nov 9;19(12):e46255. doi: 10.15252/embr.201846255 (PMC6280645; doi:10.15252/embr.201846255)
Supplement: Supplementary file 1 — Expanded View Figures PDF [file EMBR-19-e46255-s001.pdf]

Expanded View Figures

Figure EV1. Web pages of ChIP-Atlas.

A, B A snapshot of the ChIP-Atlas top page is shown in (A). From this page, users are able not only to access the four main functions of ChIP-Atlas but also to search for data of interest with a given SRX ID (A, top right) or with keywords such as antigen and cell type names (B).

C Snapshot of the Web page for the ChIP-Atlas "Peak Browser" function. Results for the settings shown are presented in Fig 2.

D Detailed information for SRX187209, including the sample metadata described by ChIP-Atlas curators and the original data submitter, processing logs, and read quality from DBCLS SRA (http://sra.dbcls.jp). Blue buttons at the top are controllers for showing the alignment and peak-call data in IGV ("View on IGV"), for downloading these data ("Download"), for viewing the analyzed data by ChIP-Atlas "Target Genes" and "Colocalization" ("View Analysis"), and for opening external pages showing details for the experimental conditions and materials ("Link Out"). This type of Web page appears on clicking the bars in the "Peak Browser" view (Fig 2) as well as by clicking SRX IDs shown in Web pages for a keyword search (B) or for "Target Genes" (Fig 3A), "Colocalization" (Fig 3B), or "Enrichment Analysis" (Fig EV3) results.

© 2018 The Authors

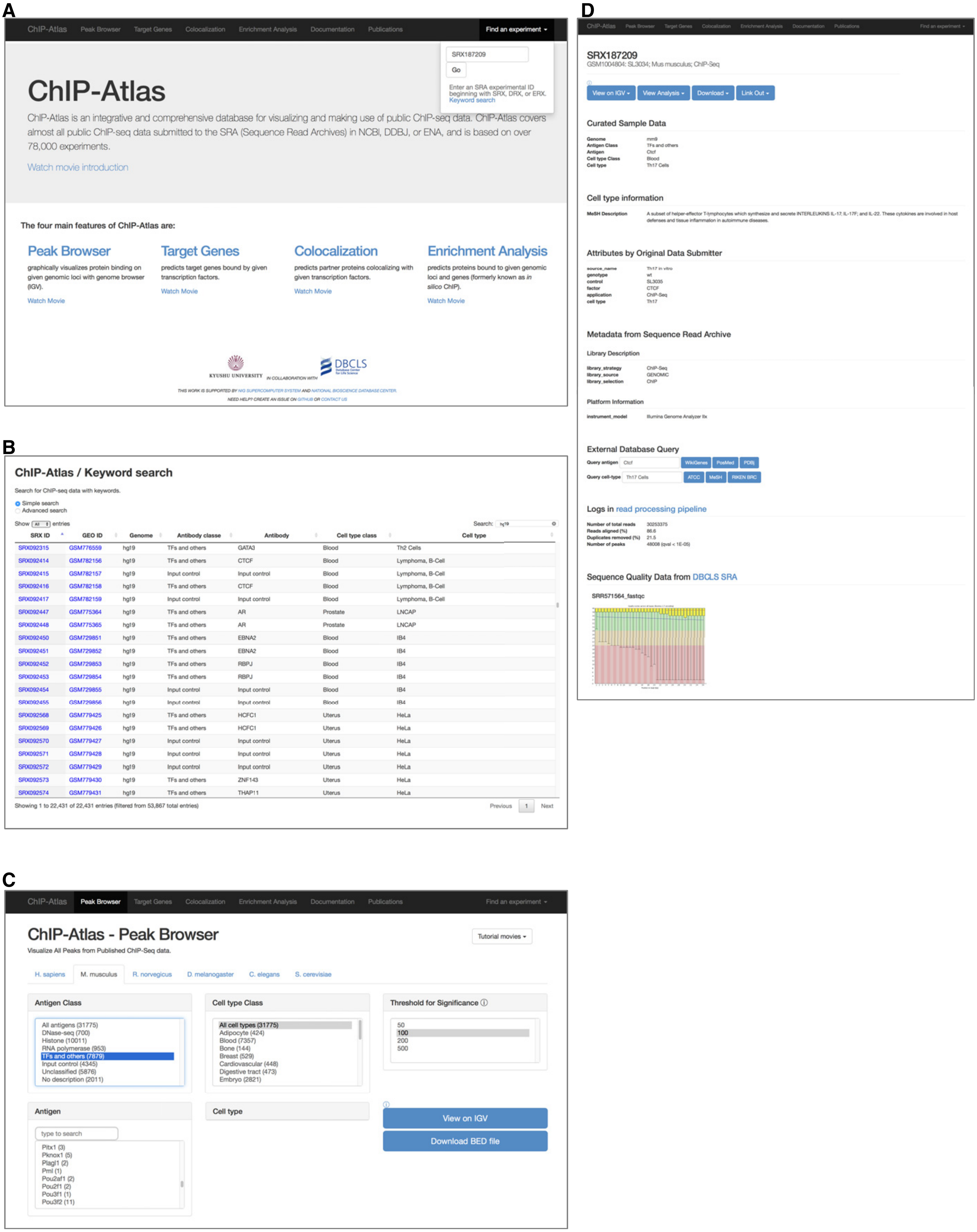

Figure EV1.

 © 2018 The Authors

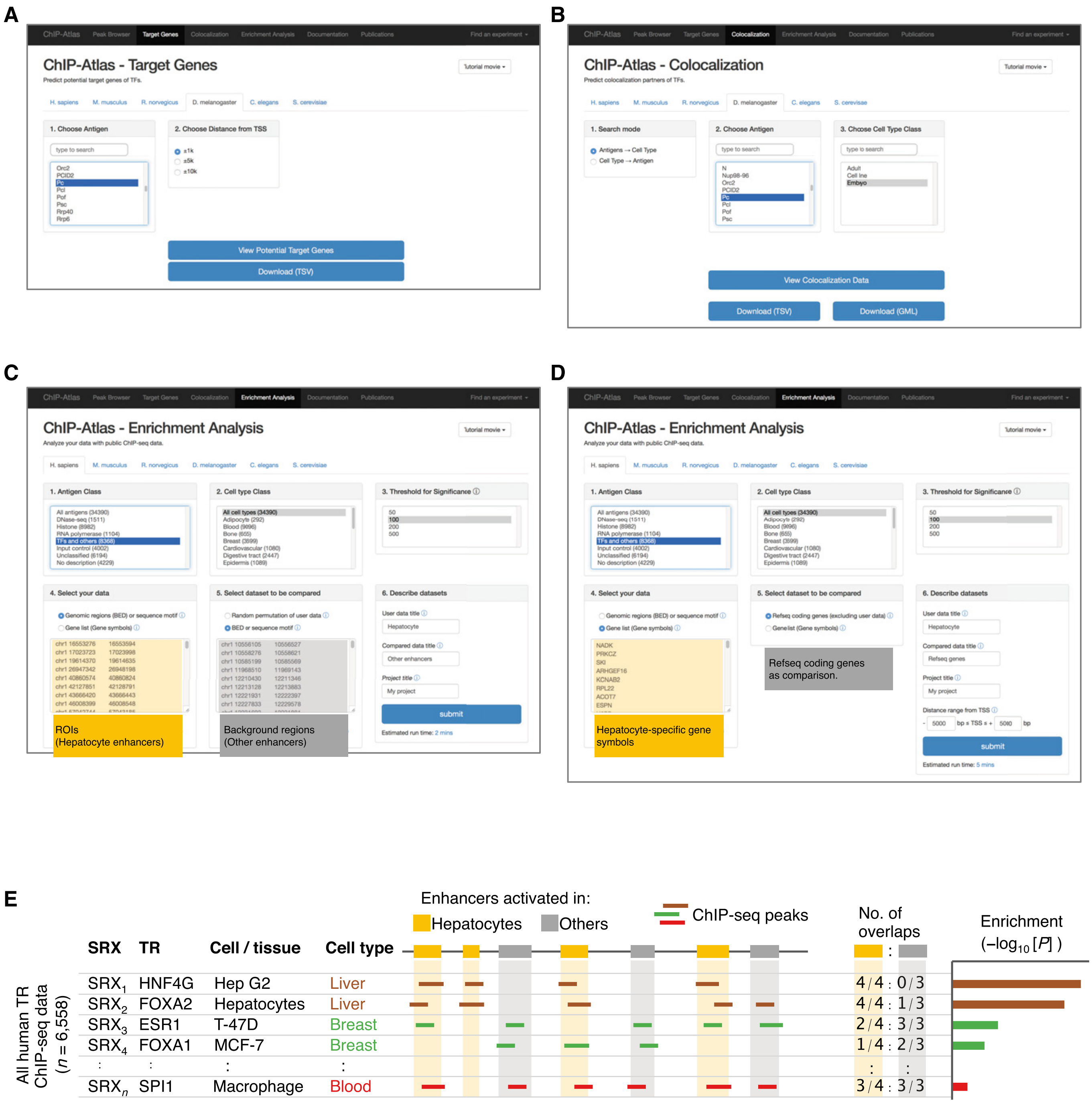

Figure EV2. Web pages for integrative analyses in ChIP-Atlas.

A, B Snapshots of Web pages for ChIP-Atlas "Target Genes" (A) and "Colocalization" (B) functions. Results for the settings shown are presented in Fig 3A and B, respectively.

C–E Snapshots of Web pages for the ChIP-Atlas "Enrichment Analysis" function with submission of genomic coordinates or gene symbols are shown in (C) and (D), respectively. Results for the settings shown are presented in Fig 4A–C and D–F, respectively. At the Web page for "Enrichment Analysis", a user can submit two sets of genomic intervals in BED format (C) or gene symbols (D): data of interest in the orange area and background data for comparison in the gray area. It is also possible to filter the results according to antigen and cell type classes as well as to set a threshold for the MACS2 score. On clicking the "submit" button, the data are sent to an NIG supercomputer server for performance of the enrichment analysis, as shown in (E). For example, on submission of BED-formatted genomic regions for hepatocyte enhancers (orange) or enhancers activated in other tissues (gray), the computational server counts the overlaps with the peaks of all SRXs (E, left). After evaluation of the significance of enrichment with Fisher's exact test (E, right), the analyzed data are returned within several minutes to the machine of the user as shown in Fig EV3.

© 2018 The Authors

Figure EV3. Examples of "Enrichment Analysis".

A, B Snapshots of the results for enrichment analysis of hepatocyte-specific enhancers with the ChIP-Atlas "Enrichment Analysis" function, for which other FANTOM5 enhancers (A) or randomly permutated regions (B) were set as background, are shown. The first row in (A), for example, indicates EP300 ChIP-seq data (SRX100544) for Hep G2 cells. The total number of peaks for EP300 is 24,334, of which 80 peaks overlap with hepatocyte-specific enhancers (n = 286) and 1,147 peaks overlap with other enhancers (n = 20,509), yielding a P-value of $1 \times 10^{-32.1}$ (Fisher's exact probability test), Q-value of $1 \times 10^{-28.3}$ (Benjamini and Hochberg method), and fold enrichment of 5.00. The table is sorted according to P-value, with HNF4A/G and FOXA1/2 in Hep G2 being ranked 3rd, 7th, 8th, 10th, and 12th. The table is also graphically summarized in Figs 4A and EV4 (top 15 and 50 experiments, respectively), in which each row of the table is represented by a bar to indicate the P-value. Note that TR peaks overlapped to a lesser extent with random background (B) than with other FANTOM5 enhancers (A).

 © 2018 The Authors

A

ID	Antigen class	Antigen	Cell class	Cell	Num of peaks	Overlaps / Hepatocyte	Overlaps / Other enhancers	Log P-val	Log Q-val	Fold Enrichment	FE > 1?
SRX100544	TFs and others	EP300	Liver	Hep G2	24334	80/286	1147/20509	-32.1	-28.3	5.00	TRUE
SRX100552	TFs and others	SP1	Liver	Hep G2	19032	64/286	755/20509	-29.7	-26.3	6.08	TRUE
SRX100449	TFs and others	HNF4G	Liver	Hep G2	15919	54/286	507/20509	-29.3	-26.0	7.64	TRUE
SRX100493	TFs and others	HEY1	Liver	Hep G2	26412	69/286	984/20509	-27.5	-24.4	5.03	TRUE
SRX100497	TFs and others	RXRA	Liver	Hep G2	13022	54/286	557/20509	-27.5	-24.4	6.95	TRUE
SRX100538	TFs and others	HDAC2	Liver	Hep G2	16071	58/286	676/20509	-27.0	-24.0	6.15	TRUE
SRX100505	TFs and others	HNF4A	Liver	Hep G2	21259	54/286	585/20509	-26.5	-23.6	6.62	TRUE
SRX100448	TFs and others	FOXA2	Liver	Hep G2	45130	67/286	1138/20509	-22.6	-19.8	4.22	TRUE
SRX150360	TFs and others	TBP	Liver	Hep G2	10293	38/286	327/20509	-21.7	-18.9	8.33	TRUE
SRX100506	TFs and others	FOXA1	Liver	Hep G2	50941	70/286	1295/20509	-21.7	-18.9	3.88	TRUE
SRX1165097	TFs and others	CREB1	Liver	Hep G2	21856	58/286	899/20509	-21.2	-18.5	4.63	TRUE
SRX100477	TFs and others	FOXA1	Liver	Hep G2	40732	66/286	1198/20509	-20.8	-18.1	3.95	TRUE
SRX150701	TFs and others	CEBPB	Liver	Hep G2	18637	52/286	849/20509	-18.0	-15.4	4.39	TRUE
SRX100545	TFs and others	JUND	Liver	Hep G2	19875	62/286	1223/20509	-17.7	-15.1	3.64	TRUE

Showing 1 to 100 of 8,010 entries Previous 1 2 3 4 5 … 81 Next

B

ID	Antigen class	Antigen	Cell class	Cell	Num of peaks	Overlaps / Hepatocyte	Overlaps / Random background	Log P-val	Log Q-val	Fold Enrichment	FE > 1?
SRX212650	TFs and others	STAT1	Blood	Monocytes-CD14+	59627	91/286	2/286	-27.6	-24.0	45.50	TRUE
SRX100544	TFs and others	EP300	Liver	Hep G2	24334	80/286	1/286	-24.9	-21.7	80.00	TRUE
SRX190321	TFs and others	MAX	Liver	Hep G2	40220	90/286	4/286	-24.8	-21.7	22.50	TRUE
SRX2770855	TFs and others	SPI1	Blood	Macrophages	88629	74/286	1/286	-22.7	-19.7	74.00	TRUE
SRX212648	TFs and others	STAT1	Blood	Monocytes-CD14+	44896	70/286	1/286	-21.3	-18.3	70.00	TRUE
SRX666556	TFs and others	HIF1A	Breast	T-47D	74508	79/286	5/286	-19.9	-17.0	15.80	TRUE
SRX212649	TFs and others	STAT1	Blood	Monocytes-CD14+	24923	66/286	1/286	-19.8	-17.0	66.00	TRUE
SRX100493	TFs and others	HEY1	Liver	Hep G2	26412	69/286	2/286	-19.6	-16.8	34.50	TRUE
SRX1023792	TFs and others	SPI1	Blood	Macrophages	114884	74/286	4/286	-19.1	-16.5	18.50	TRUE
SRX100552	TFs and others	SP1	Liver	Hep G2	19032	64/286	1/286	-19.1	-16.5	64.00	TRUE
SRX190269	TFs and others	SP1	Lung	A549	29434	64/286	1/286	-19.1	-16.5	64.00	TRUE
SRX100448	TFs and others	FOXA2	Liver	Hep G2	45130	67/286	2/286	-18.9	-16.3	33.50	TRUE
SRX100506	TFs and others	FOXA1	Liver	Hep G2	50941	70/286	3/286	-18.8	-16.3	23.33	TRUE
SRX100403	TFs and others	NR3C1	Lung	A549	18382	58/286	0/286	-18.6	-16.1	99999	TRUE

Showing 1 to 100 of 4,352 entries Previous 1 2 3 4 5 … 44 Next

Figure EV3.

Figure EV4. Results of enrichment analysis for tissue-specific enhancers.
The results of enrichment analysis for FANTOM5 tissue-specific enhancers are sorted according to the minimum *P*-value (P_{min}) for each facet. The bar charts indicate *P*-value (horizontal axis) for the top 50 enriched ChIP-seq experiments (vertical axis), with the colors denoting cell type classes according to the color palette (bottom right).

 © 2018 The Authors

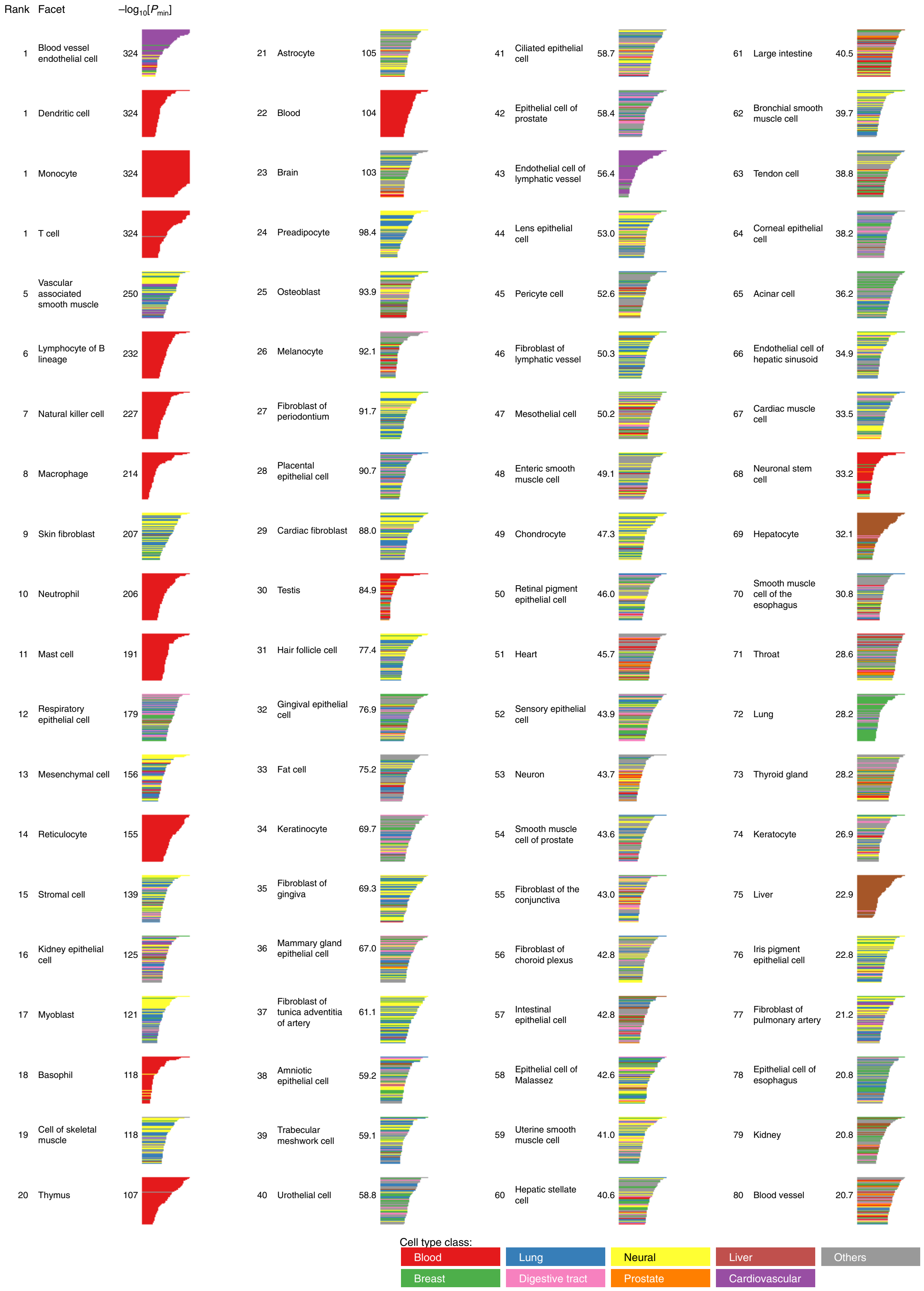
Rank Facet −log10[Pmin]
1 Blood vessel endothelial cell 324
1 Dendritic cell 324
1 Monocyte 324
1 T cell 324
5 Vascular associated smooth muscle 250
6 Lymphocyte of B lineage 232
7 Natural killer cell 227
8 Macrophage 214
9 Skin fibroblast 207
10 Neutrophil 206
11 Mast cell 191
12 Respiratory epithelial cell 179
13 Mesenchymal cell 156
14 Reticulocyte 155
15 Stromal cell 139
16 Kidney epithelial cell 125
17 Myoblast 121
18 Basophil 118
19 Cell of skeletal muscle 118
20 Thymus 107
21 Astrocyte 105
22 Blood 104
23 Brain 103
24 Preadipocyte 98.4
25 Osteoblast 93.9
26 Melanocyte 92.1
27 Fibroblast of periodontium 91.7
28 Placental epithelial cell 90.7
29 Cardiac fibroblast 88.0
30 Testis 84.9
31 Hair follicle cell 77.4
32 Gingival epithelial cell 76.9
33 Fat cell 75.2
34 Keratinocyte 69.7
35 Fibroblast of gingiva 69.3
36 Mammary gland epithelial cell 67.0
37 Fibroblast of tunica adventitia of artery 61.1
38 Amniotic epithelial cell 59.2
39 Trabecular meshwork cell 59.1
40 Urothelial cell 58.8
41 Ciliated epithelial cell 58.7
42 Epithelial cell of prostate 58.4
43 Endothelial cell of lymphatic vessel 56.4
44 Lens epithelial cell 53.0
45 Pericyte cell 52.6
46 Fibroblast of lymphatic vessel 50.3
47 Mesothelial cell 50.2
48 Enteric smooth muscle cell 49.1
49 Chondrocyte 47.3
50 Retinal pigment epithelial cell 46.0
51 Heart 45.7
52 Sensory epithelial cell 43.9
53 Neuron 43.7
54 Smooth muscle cell of prostate 43.6
55 Fibroblast of the conjunctiva 43.0
56 Fibroblast of choroid plexus 42.8
57 Intestinal epithelial cell 42.8
58 Epithelial cell of Malassez 42.6
59 Uterine smooth muscle cell 41.0
60 Hepatic stellate cell 40.6
61 Large intestine 40.5
62 Bronchial smooth muscle cell 39.7
63 Tendon cell 38.8
64 Corneal epithelial cell 38.2
65 Acinar cell 36.2
66 Endothelial cell of hepatic sinusoid 34.9
67 Cardiac muscle cell 33.5
68 Neuronal stem cell 33.2
69 Hepatocyte 32.1
70 Smooth muscle cell of the esophagus 30.8
71 Throat 28.6
72 Lung 28.2
73 Thyroid gland 28.2
74 Keratocyte 26.9
75 Liver 22.9
76 Iris pigment epithelial cell 22.8
77 Fibroblast of pulmonary artery 21.2
78 Epithelial cell of esophagus 20.8
79 Kidney 20.8
80 Blood vessel 20.7
Cell type class:
Blood
Lung
Neural
Liver
Others
Breast
Digestive tract
Prostate
Cardiovascular

Figure EV4.

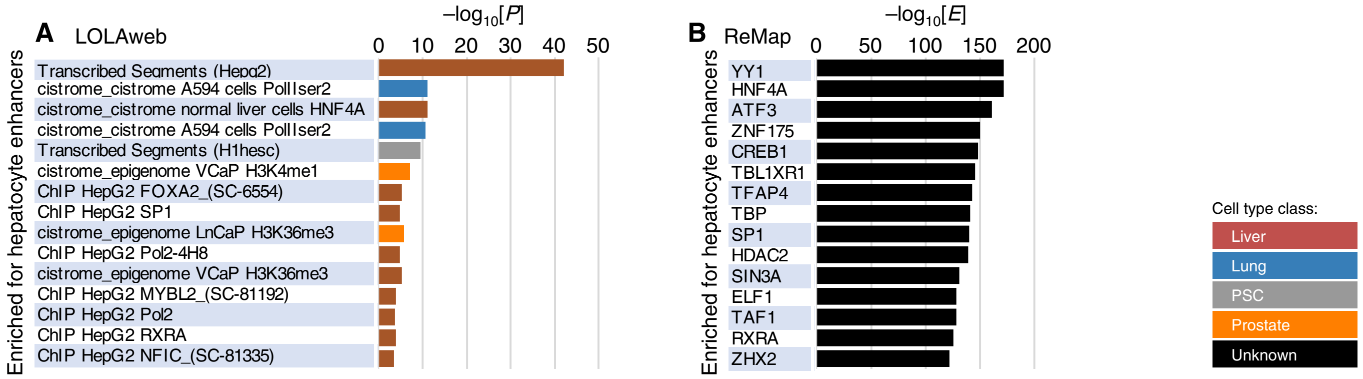

Figure EV5. Results of enrichment analysis with other tools.

A, B Enrichment analysis for hepatocyte enhancers was performed with LOLAweb (A), for which other FANTOM5 enhancers were used as background, and with "Annotation Tool" of ReMap (B), for which only random regions were available as background. The top 15 ChIP-seq experiments showing statistical significance are shown as bar charts.

 © 2018 The Authors
